# Tear meniscus height comparison between AS-OCT and Oculus Keratograph® K5M

**DOI:** 10.22336/rjo.2024.72

**Published:** 2024

**Authors:** Angeline Lim Pei Yik, Fakhruddin Shamsheer Barodawala

**Affiliations:** Faculty of Optometry and Vision Sciences, SEGi University, Selangor, Malaysia

**Keywords:** tear meniscus height, dry eyes, OCT, keratograph, agreement, CL = Contact Lens, DED = Dry eye disease, NIKBUT = Non-invasive keratographic breakup time, OCT = Optical Coherence Tomography, TMH = Tear meniscus height

## Abstract

**Objectives:**

To evaluate the agreement between Spectral-Domain Optical Coherence Tomography (AS-OCT) and Oculus Keratograph® 5M in measuring tear meniscus height (TMH) and to assess the impact of contact lens wear on these measurements. **Materials and methods**: TMH was measured in 54 healthy eyes using AS-OCT (3D OCT-1 Maestro, Topcon, Tokyo, Japan) and Oculus Keratograph® 5M (OCULUS Optikgeräte, Wetzlar, Germany), with and without contact lens wear. Bland-Altman analysis was used to assess agreement between the two devices. Delefilcon A, water-gradient, daily disposable contact lenses were used, and measurements were carried out after a 20-minute adaptation period.

**Results:**

The means of TMH without the contact lenses were 0.21 ± 0.06 mm and 0.20 ± 0.05 mm obtained from AS-OCT and Oculus K5M, respectively, and these measurements were not statistically significant (t (53) = 0.99, p = 0.33). No significant differences were observed in TMH compared to contact lenses (t (53) = 1.52, p = 0.13). Agreement between measurements obtained by both the instruments was assessed using Bland-Altman analysis. The limits of agreement were within clinically acceptable ranges (0.10 mm - 0.15 mm), with no evidence of significant bias (t = -0.32, r = 0.22). The results obtained with contact lenses were also not statistically significant (t (53) = 1.52, p < 0.05).

**Discussion:**

The present study compared tear meniscus height (TMH) measurements obtained from AS-OCT and Oculus K5M in subjects with and without contact lens wear. Both instruments showed good agreement, with AS-OCT consistently measuring slightly higher TMH values than Oculus K5M. The mean TMH values were similar to those of previous studies, indicating normal tear film in the subjects. Contact lens wear was found to reduce TMH slightly, but it returned to baseline after a short adaptation period. The Bland-Altman analysis confirmed good agreement between the two instruments, with most data points falling within the limits of agreement. These findings suggest that AS-OCT and Oculus K5M can be reliable tools for measuring TMH and can be used interchangeably for clinical practice.

**Conclusion:**

AS-OCT and Oculus Keratograph® 5M showed comparable results in measuring TMH, suggesting potential interchangeability in clinical practice. Further validation in broader clinical settings and diverse subject groups may be warranted.

## Introduction

The tear film is essential for maintaining ocular comfort and optimal visual function [1]. The tear meniscus is the collection of tears found at the junction of the bulbar conjunctiva and the margins of both eyelids. Tear meniscus height (TMH) is a valuable biomarker for diagnosing aqueous-deficient dry eye [2]. It holds 75-90% of the total tear volume, and tears are applied to the precorneal tear film [3]. Based on the Tear Film & Ocular Surface Society Disease Early Warning System (TFOS DEWS II), TMH is directly related to the lacrimal secretory rate. A consistently stable precorneal tear film has been considered a fundamental aspect of ocular health [4].

Tear film assessment is crucial for diagnosing dry eye disease (DED). Various methods exist, with meniscometry, focusing on tear meniscus volume, being one approach. However, factors like blinking, measurement location, and environmental conditions influence their accuracy. Quantitative tear meniscus height, curvature, and area assessment offer improved diagnostic precision. Still, it relies on operator expertise and is affected by factors such as fluorescein staining and time since blinking. Slit lamp meniscometry and Schirmer’s test have limitations in terms of repeatability and diagnostic accuracy [5].

Spectral-domain optical coherence tomography (OCT) (3D OCT-1 Maestro (Topcon, Tokyo, Japan)) provides high-resolution, noninvasive imaging of ocular tissues, including the tear meniscus. Its ability to generate three-dimensional images offers a precise alternative to traditional meniscometry for measuring the tear meniscus height (TMH) and is recognized as an accurate alternative [1,6].

The Oculus Keratograph® 5M (manufactured by OCULUS Optikgeräte, Wetzlar, Germany) is a corneal topographer device based on the Placido disc principle. This equipment features a modified tear film scanning function. Despite being an invasive technique, it can accurately represent the actual condition of the ocular surface. It serves as a straightforward approach for screening dry eye [7].

AS-OCT is widely used in clinical practice due to its non-invasive nature and ability to generate detailed, three-dimensional images of ocular structures, including the tear meniscus [3,8]. The Oculus K5M is a valuable tool for assessing the anterior eye and tear film, including tear film quality. Its advantages are that it does not require direct contact with the lower eyelid and can provide a broader view of the entire eye to measure TMH.

This study compares the TMH measurements obtained using AS-OCT and Oculus K5M in subjects with and without contact lens wear. Given the widespread availability of AS-OCT in clinical settings compared to the Oculus K5, this study seeks to determine if AS-OCT can be a reliable alternative for measuring TMH and if the values obtained from both instruments are comparable [8].

## Materials and methods

This study included healthy adult participants aged 18-30 without ocular pathologies. Subjects were divided into contact lens wearers and non-contact lens wearers. Contact lens wearers were required to abstain from lens wear for 24 hours before the study.

A cross-sectional study design was employed to compare TMH measurements obtained using AS-OCT and Oculus K5M. Participants were randomly assigned to determine the order of instrument use. TMH measurements were initially taken in both eyes without contact lenses. Subsequently, a Delefilcon A (Dailies Total 1) contact lens was applied to the right eye, and measurements were repeated after a 20-minute adaptation period. All measurements were performed within the same timeframe under standardized conditions to minimize bias.

To minimize bias, TMH measurements were performed in a randomized order on each subject within the same day period [2]. Based on the earlier study, the measurements were made below the corneal vertex at the cornea’s 6 o’clock position with the lower eyelid. The method employed in the present study for measuring TMH using AS-OCT and Oculus K5M was like that described in the previous research, with consistent imaging conditions and standardized measurement procedures.

**Fig. 1:**
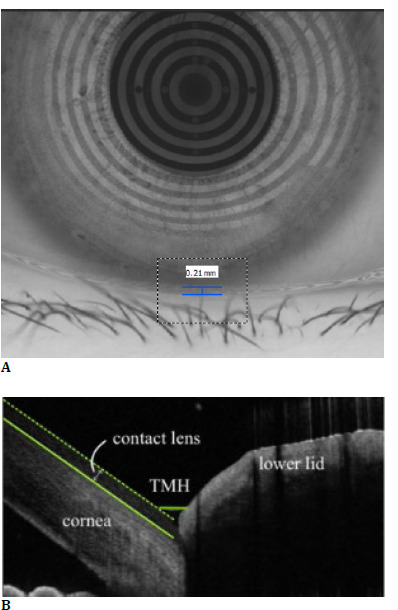
Tear meniscus height measurement examples using (**A**) Oculus Keratograph 5M and (**B**) AS-OCT

Imaging scans using AS-OCT and Oculus K5M were conducted under dim lighting and similar humidity. All assessments were consistently performed at the same time of day. Subjects were instructed to begin by measuring tear meniscus height through image scans with AS-OCT and Oculus K5M by the sequences determined by the drawing of lots. In the case of AS-OCT, a vertical scan beam was used to image the tear meniscus positioned between the cornea and the eyelid’s tear boundary line. For the Oculus K5M, lower TMH images were taken and assessed at the central position relative to the pupil centre, using a built-in ruler, as shown in **Fig. 1 A.** The scanning line’s length was 6 mm, and it was directed towards the 6 o’clock position of the cornea, as shown in **Fig. 1 B**.

The University’s Ethics Committee approved the study, and informed consent was obtained from all participants.

## Results

This study included 54 healthy right eyes (26 males, 28 females). The mean age of the subjects was 22.87 ± 2.45 years (ranging from 19 to 31 years old). **Table 1** presents descriptive statistics for TMH measurements obtained using AS-OCT and Oculus K5M, both with and without contact lenses. Data were typically distributed based on the Shapiro-Wilk test.

**Table 1:** Descriptive data of TMH measurements using AS-OCT and Oculus K5M

TMH	Mean ± Std Deviation (mm)
AS-OCT	0.21 ± 0.06
Oculus K5M	0.20 ± 0.05
AS-OCT with CL	0.19 ± 0.06
Oculus K5M with CL	0.18 ± 0.04

The paired t-test showed no significant difference in tear meniscus height (TMH) measurements between AS-OCT and Oculus K5M without contact lenses (t (53) = 0.99, p = 0.33). Furthermore, there were no significant differences in TMH when comparing OCT and Oculus K-5 measurements with contact lenses (t (53) = 1.52, p = 0.13).

Moreover, there were no significant differences in tear meniscus height (TMH) when using OCT with and without contact lenses (t (53) = 1.23, p = 0.22). However, a significant difference in TMH was seen when using Oculus K-5 with and without contact lenses (t (53) = 2.27, p < 0.05).

**Fig. 2:**
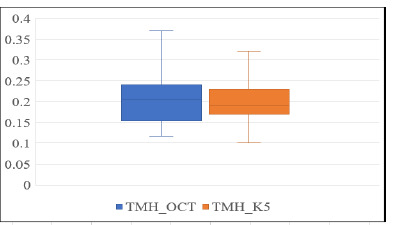
Boxplot showing TMH measurements with AS-OCT and Oculus K5M

According to the boxplot in **Fig. 2** above, no outlier data points were detected within the displayed range for AS-OCT or Oculus K5M measurements with TMH measurements without the contact lenses.

**Fig. 3:**
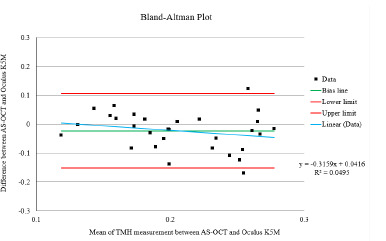
The Bland-Altman plot of the difference between the mean of AS-OCT and Oculus K5M. The Green line indicates bias, the red line indicates the upper and lower limits of agreement (LoA), and the blue line indicates linear regression.

A Bland-Altman plot was constructed to assess the agreement between AS-OCT and Oculus K5M for TMH measurements without contact lenses (**Fig. 3**). The mean difference (bias) was -0.02 mm, with limits of agreement of -0.15 mm and 0.10 mm. No proportional bias was observed (r = 0.22).

## Discussion

The present study investigated the tear meniscus height (TMH) measurements using AS-OCT and Oculus K5M in subjects with and without contact lens wear. The primary objective was to determine the agreement between TMH measurements obtained from these two instruments. It is essential to differentiate between absolute TMH and reflex tear TMH. While slit lamp measurements capture reflex tear TMH, non-invasive tools like AS-OCT and Oculus K5M provide values closer to absolute TMH, which are generally higher [9]. This might explain the tendency of the Oculus Keratograph to record higher TMH values compared to slit lamp measurements. The Oculus Keratograph® 5M (OCULUS Optikgeräte, Wetzlar, Germany) uses infrared imaging. At the same time, the AS-OCT (3D OCT-1 Maestro, Topcon, Tokyo, Japan) employs non-invasive, high-resolution, low-coherence interferometry to image the eye’s anterior surface. The differences in illumination systems may contribute to variability in TMH measurements [2].

A previous study reported that mean TMH values above 0.20 mm were observed in non-clinical participants, and reproducibility and repeatability were demonstrated among different observers [10,11]. The present study showed that the mean TMH value was 0.20 ± 0.05 mm and 0.21 ± 0.06 mm for Oculus K5M and AS-OCT, respectively, without the contact lens. This study showed that TMH measured with Oculus K5M was slightly lower than the AS-OCT, in which both instruments are significantly correlated. A previous study reported that Oculus K5M was lower by 0.01 mm than FD-OCT amongst healthy participants [2].

Another study investigated the mean TMH measurements, which were 0.29 ± 0.16 mm for Oculus K5M and 0.27 ± 0.16 mm for AS-OCT in non-clinical participants [6,12]. The mean TMH value for Medmont Meridia was 0.24 ± 0.09 mm. From the comparison of TMH values, it can be concluded that Medmont Meridia shows good agreement with AS-OCT, as there were no statistically significant differences between them. However, there is a substantial difference between the TMH measurements obtained by Medmont Meridia and Oculus Keratograph 5 M, with Oculus Keratograph 5 M showing higher values [2].

In another study done by Wei et al. (2012), the mean TMH values in the dry eye group, as measured by Oculus Keratograph 4 and AS-OCT, were found to be 0.24 ± 0.07 mm and 0.25 ± 0.08 mm whereas 0.31 ± 0.04 mm and 0.32 ± 0.12 mm in non-dry eye groups respectively [9]. They concluded that the results of Oculus Keratography 4 of TMH values significantly correlated with AS-OCT. Another study reported that the TMH was obtained 3-4 seconds after an average blink to analyze the accuracy of the TMH by using different techniques. The TMH measurement using the slit lamp microscope with reticule was 0.20 ± 0.05 mm and 0.22 ± 0.01 mm, and the OCT measurements were 0.24 ± 0.07 mm. TMH measurements using AS-OCT are higher than those measured using slit-lamp [13].

The TFOS DEWS II Report recommended that the cut-off values for AS-OCT TMH values be 0.22 mm or 0.24 mm to discriminate between normal and abnormal TMH [14]. However, baseline TMH values in regular groups were 0.20 ± 0.05 mm using Oculus K5M [13]. Therefore, based on previous studies’ TMH measurements, the present study indicated that the subjects selected had no signs of dry eye disease.

Prevalence rates of Dry Eye Disease (DED) vary from 5% to 50%, reaching up to 75% in adults over 40, with women most affected [15]. A Japanese study found that contact lens wearers are 2.38 times more likely to be diagnosed with dry eye and 3.61 times more likely to experience severe symptoms than non-wearers. Patients with Contact Lens Associated Dry Eye (CLADE) exhibit distinct ocular characteristics, including Lid Wiper Epitheliopathy (LWE) in 80% of CLADE patients versus 13% of asymptomatic contact lens users [16].

After wearing the contact lenses for 20 minutes, the TMH values returned to baseline [17]. Furthermore, TMH values remained stable for at least 4 hours after wearing the contact lenses. Thus, this suggests that tear film adjusted to the presence of the contact lenses maintained a consistent level of tears around the eyes [17]. In line with the present study, there were no notable differences in TMH measurements obtained using AS-OCT with and without contact lenses, as a 20-minute adaptation period was provided to the subjects before the TMH assessment.

The present study showed that the TMH was reduced by approximately 0.02 mm (20 µm) with contact lens wear. This is supported by another previous study that reported that initially, the pre-contact lens tear film is thicker due to reflex tearing and increased tear production. However, once the reflex tearing subsides, the thickness of the pre-contact lens tear film decreases to approximately 2 micrometers [18].

The Bland-Altman analysis demonstrated good agreement between AS-OCT and Oculus K5M for TMH measurements, with a consistent negative bias of -0.02 mm. While two data points fell outside the limits of agreement, the two instruments exhibited comparable results overall. This is supported by previous research showing a good correlation between Oculus K5M and AS-OCT in dry eye patients. The AS-OCT yielded higher values compared to Oculus K5M. By showing that their differences were only 0.02 mm consistently, the mean difference being close to zero indicated that most data points from the two instruments were similar findings.

Bland-Altman plots were also used to determine the limits of agreement, and most data points fell within the limit of agreement. Two data points fell outside the boundaries of agreement, indicating differences between these measurements. A Bland-Altman plot demonstrates these techniques’ comparability and suggests no evidence of proportional bias.

Considering the two outliers on the Bland-Altman plot, it was found that either the Oculus K5M or the AS-OCT could overestimate the measurements. Instead, these deviations can likely be attributed to random measurement errors or specific variations in the image acquisition process. Despite these two outliers, the Bland-Altman analysis indicated no statistically significant difference between the measurement methods [19].

This previous study investigated dry eye disease subjects and resulted in TMH measurements with Oculus K5M closely correlating with AS-OCT, with good consistency [9,10]. This means that Oculus K5M provides results that agree with the results obtained from AS-OCT, which can also prove that it can be reliably used as an alternative tool for similar measurements. In addition, the Bland-Altman analysis concluded that they can be used interchangeably for TMH measurement.

## Conclusion

This study demonstrated a strong agreement between AS-OCT and Oculus K5M in measuring tear meniscus height (TMH) within a controlled study population. These findings suggest that both devices can be reliably used interchangeably for TMH assessment in clinical practice. To further solidify the interchangeability of these instruments, future studies should explore their performance in a broader range of patient populations and clinical settings.

## References

[1] Canan H, Altan-Yaycioglu R, Ulas B, Sizmaz S, Coban-Karatas M (2014). Interexaminer reproducibility of optical coherence tomography for measuring the tear film meniscus. Curr Eye Res.

[2] oares I, Ramalho E, Brardo FM, Fernandes A, Ramalho E, Brardo FM (2024). Tear meniscus height agreement and reproducibility between two corneal topographers and spectral-domain optical coherence tomography. Clin Exp Optom.

[3] Raj A, Dhasmana R, Nagpal RC (2016). Anterior Segment Optical Coherence Tomography for Tear Meniscus Evaluation and its Correlation with Other Tear Variables in Healthy Individuals. J Clin Diagn Res.

[4] Craig JP, Nichols KK, Akpek EK, Caffery B, Dua HS, Joo C-K (2017). TFOS DEWS II Definition and Classification Report. Ocul Surf.

[5] Wolffsohn JS, Arita R, Chalmers R, Djalilian A, Dogru M, Dumbleton K (2017). TFOS DEWS II Diagnostic Methodology report. Ocul Surf.

[6] Eroglu FC, Karalezli A, Dursun R (2016). Is optical coherence tomography an effective device for evaluation of tear film meniscus in patients with acne rosacea?. Eye.

[7] Tian L, Qu JH, Zhang XY, Sun XG (2016). Repeatability and Reproducibility of Noninvasive Keratograph 5M Measurements in Patients with Dry Eye Disease. J Ophthalmol.

[8] Wan C, Hua R, Guo P, Lin P, Wang J, Yang W (2023). Measurement method of tear meniscus height based on deep learning. Front Med.

[9] Wei A, Le Q, Hong J, Wang W, Wang F, Xu J (2016). Assessment of Lower Tear Meniscus. Optom Vis Sci.

[10] Baek J, Doh SH, Chung SK (2015). Comparison of tear meniscus height measurements obtained with the keratograph and Fourier domain optical coherence tomography in dry eye. Cornea.

[11] Wong SJ, Barodawala FS, Azmi ANH (2022). In Vivo Wettability of HydraGlyde® Silicone Hydrogel Lens With and Without HydraGlyde® Containing Lens Care Solution. J Dry Eye Dis.

[12] Popovici DM, Banc A (2021). Tear evaluation by anterior segment OCT in dry eye disease. Rom J Ophthalmol.

[13] Koh S, Ikeda C, Watanabe S, Oie Y, Soma T, Watanabe H (2015). Effect of non-invasive tear stability assessment on tear meniscus height. Acta Ophthalmol.

[14] Niedernolte B, Trunk L, Wolffsohn JS, Pult H, Bandlitz S (2021). Evaluation of tear meniscus height using different clinical methods. Clin Exp Optom.

[15] Mondal H, Kim HJ, Mohanto N, Jee JP (2023). A Review on Dry Eye Disease Treatment: Recent Progress, Diagnostics, and Future Perspectives. Pharmaceutics.

[16] Kojima T (2018). Contact lens-associated dry eye disease: Recent advances worldwide and in Japan. Investig Ophthalmol Vis Sci.

[17] Chen Q, Wang J, Shen M, Cui L, Cai C, Li M (2011). Tear menisci and ocular discomfort during daily contact lens wear in symptomatic wearers. Investig Ophthalmol Vis Sci.

[18] Guillon M, Theodoratos P, Patel K, Gupta R, Patel T (2019). Pre-contact lens and pre-corneal tear film kinetics. Contact Lens Anterior Eye.

[19] Edgar AK, Connor HRM, Kamarelddin S, Musich J, Mclouta S, Choi E (2024). Anterior segment optical coherence tomography meibography compared with keratograph meibography. Ophthalmic Physiol Opt.

